# Neurology Telemusic Program at the Time of the COVID-19 Pandemic: Turning Hospital Time Into Aesthetic Time During Crisis

**DOI:** 10.3389/fneur.2021.749782

**Published:** 2021-12-13

**Authors:** Borna Bonakdarpour, Alyssa McFadden, Skye Zlotkowski, Daniel Huang, Michelle Shaker, Bailey Shibata, William Haben, Charlinda Brashear, Anny Sandoval, Carianne Breitenbach, Caren Rodriguez, Jennifer Viamille, Mark Porter, Kristin Galic, Michelle Schaeve, Daniel Thatcher, Clara Takarabe

**Affiliations:** ^1^The Ken and Ruth Davee Department of Neurology, Feinberg School of Medicine, Northwestern University, Chicago, IL, United States; ^2^Department of Neurology, Northwestern Medicine, Chicago, IL, United States; ^3^Department of Psychiatry, Riveredge Hospital, Forest Park, IL, United States; ^4^Department of Food Science and Human Nutrition, University of Illinois Urbana-Champaign, Champaign, IL, United States; ^5^Department of Biology, Loyola University Chicago, Chicago, IL, United States; ^6^Feinberg School of Medicine, Northwestern University, Chicago, IL, United States; ^7^Department of Recreation Therapy, Jesse Brown Veterans Affair Medical Center, Chicago, IL, United States

**Keywords:** telemusic, music intervention, non-pharmacological intervention, COVID-19, psychological first aid

## Abstract

Strict precautions during the COVID-19 pandemic left patients isolated during already stressful hospital stays. Research indicates that listening to music recruits regions in the brain involved with social interaction and reduces feelings of loneliness. We formed a team of clinicians and clinical musicians to bring music to the bedside, as “psychological first aid.” Our goal was to reduce feelings of anxiety and isolation in patients admitted to the Northwestern Memorial Hospital's neurosciences unit. Participants were offered 30–40-min live music sessions over FaceTime by a violist in consultation with a music therapist and a certified music practitioner. Music used for the interventions was personalized. Participants were evaluated with the Music Assessment Tool where they indicated their musical preferences and music to which they objected. Following the intervention, participants answered a questionnaire assessing how music impacted their emotional state based on a 1–10 Likert scale. Scores were then averaged across all patients and were calculated as percentages. Eighty-seven sessions were completed during a 3-month period. Despite different degrees of disability, most patients engaged aesthetically with the music. The likelihood to recommend (LTR) for the program was 98%; participants tended to highly agree that the intervention improved their emotional state (92%); that it provided a pleasurable experience (92.4%); and that it reduced their stress and anxiety (89.5%). This pilot project showed that the telemusic intervention was feasible for our neurosciences patients during the COVID-19 pandemic. Our results are consistent with previous in-person hospital-based music interventions and highlight the importance of such programs when in-person interventions are not possible. This pilot project serves as a prelude to further investigate mechanisms by which music interventions can support admitted neurology patients.

## Background

COVID-19 arrived in the United States in January of 2020. The first community-based cases were documented in early March and by mid-March rapid spread of the virus prompted some states to issue stay-at-home orders. Logistical issues to contain the infection, including the shortage of essential supplies, travel restrictions, business closures, individual factors such as inability to follow rules and regulations, and insufficient information about the virus created significant psychological distress as the number of cases skyrocketed (1). By mid-March, following increasing cases in Illinois, strict stay-at-home orders were issued (2). In hospitals, mandatory precautions left many patients isolated and anxious (3).

Psychological first aid (PFA) is an evidence-informed approach to assist individuals in the aftermath of disasters (4). PFA is delivered by disaster response workers who provide early assistance, including mental health professionals, religious professionals, disaster volunteers, and qualified music practitioners (5). Music interventions influence physiological responses, behaviors, cognition, memory, and emotion. Music provides a non-verbal means to establish self-regulation and reduce a stressed physiological state (5, 6).

In an effort to confront the effects of isolation, health care professionals developed creative ways to keep patients connected with their families and friends. There is a convincing body of evidence that suggests that music can be used as an intervention to help with symptoms of anxiety and loneliness (7–10). Music has also been widely used before and after procedures in hospitals (11–13) and for neurologic diseases (14). There is a convergence between physiologic state and music-related emotional experience that is neurophysiologically determined (15). Pathways of music processing are in close communication with the autonomic, emotional, and social systems which influence heart rate, respiratory rate, and muscles of facial expression (16).

Through the dopaminergic pathway in the brain, music relieves feelings of tension and brings pleasure (17). Music *qua* PFA aided in critical circumstances such as Hurricane Katrina, war in Bosnia, and Sierra Leone (18, 19). The American Music Therapy Association has called music “psychological first aid” for disaster relief (5). We assembled a team of clinicians and clinically trained musicians to help patients who were admitted to the neurology unit at Northwestern Memorial Hospital during the early months of the COVID-19 pandemic.

Music interventions for hospitalized patients with neurologic disorders require special considerations. Neurologic patients have a wide range of disabilities due to nervous system pathology affecting them both physically and cognitively. Patients may have challenges with reception and expression of musical ideas. In some patients, music can cause adverse reactions (20). At the same time, as a non-verbal way of communication, music can evoke responses when verbal communication fails (21, 22).

By April 2020, no instituted protocol was available regarding strategies to bring music to the bedside within our neurosciences unit. While some individual music therapists have been using virtual platforms to provide music therapy services as early as 2018, it did not become common practice until the pandemic arrived in the spring of 2020. Music therapists began to explore telemusic therapy as a viable option to provide services to those populations who would be put at great risk with in-person sessions (23).

The goal of this feasibility study was to bring music to the bedside in a neurosciences unit as PFA. We also planned to assess whether such intervention in the form of telemedicine would be viable. Considering research indicated that the interactivity of live music was more strongly associated with positive outcomes (24–26), we chose live, over recorded music. Due to mandatory COVID-19 precautions in the hospital, musical activities were organized virtually. Floor coordinators were available in person and helped with recruitment, coordination of telemusic intervention, and assessment.

## Methodology

Participants were offered 30–40-min live music sessions over FaceTime by a viola player in consultation with a music therapist and a certified music practitioner. Pitches between middle C (262 Hz) and one octave higher (524 Hz) stimulate the social engagement network and confer a sense of safety to the listener. Music in this frequency band elicits visceral and emotional states that are not associated with feelings of doom or urgency (15). For this reason, the viola was chosen as the intervention instrument.

The material used for interventions was personalized by using familiar or improvised music depending on the patient's condition. Personalization was accomplished using a standardized questionnaire to avoid pieces that could negatively affect the participants. Immediately following the intervention participants filled out a questionnaire assessing how they felt the intervention impacted their general emotional state, feelings of tension, and loneliness.

### Telemusic Team

In order to evaluate, enroll participants, and plan the music delivered to the patients, we put together a team consisting of nurse/program managers (CR, JV, MS, KG, SC), floor coordinators (a team of 4 social workers (SW), a pre-med intern: (MS, BS, CB, WH, SZ), a music practitioner intern (CT), a music therapist (AM), a certified music practitioner consultant (DT), who were led by a cognitive neurologist (BB). All team members had gone through HIPAA training and operated within the bounds of privacy regulations. We had approval from the Northwestern Institutional Review Board (IRB) to retrospectively study clinical outcomes of patient interventions.

### Technology

The music practitioner intern (violist) used a MacBook Air connected to Wi-Fi and performed using a *Blue Yeti Nano Professional Condenser USB Microphone*. At the bedside, an iPad connected to Wi-Fi and secured on a stand was used to communicate with the patient with the assistance of floor SWs. Pre- and post-evaluations were done on the same iPad. Our team used Sentact for data collection and reporting (https://sentact.com/). Sentact is a digital rounding platform used to collect patient feedback during their hospitalization.

### Enrollment Process

The neuroscience floor at NMH consists of general neurology, neurosurgery, step-down, and epilepsy monitoring (EMU) beds from which the participants were enlisted into the program. A few patients from the neurology infusion suite were included in the study. To identify patient appropriateness for the intervention, the floor coordinators leveraged the use of the Patient Assignment schedule available daily at the nurses' desk. Coordinators consisted of 4 SWs and a pre-med intern.

SWs provided an information sheet about the program, evidence supporting such intervention, and asked whether the patients would be interested in a telemusic intervention. Patients who were interested consented to participate in this study and filled out the Music Assessment Tool (27) (MAT). In the preliminary section of the MAT, it documented the patient's name, date, diagnosis, age, level of education, vocation, ethnic background, and religion. This section also documented the reason for admission as well as emotionally significant events prior to admission into the hospital, the current mood state, and whether the patient's hearing was impaired.

Participants selected their musical genres of interest and specified groups/bands they liked. In addition, participants also identified music and groups they disliked avoiding adverse reactions. Musical genres included classical, opera, jazz, classic rock, rock, Christian rock, sacred (gospel and hymns), rhythm and blues (R&B), pop, movie music, folk, musicals, country, world, adult contemporary music.

SWs interviewed patients who were interested in telemusic intervention. All COVID-19-related precautions were followed during the encounter with the participant (28). The SWs also made the schedule for when the patients would have their telemusic intervention. These assessments were sent to the neurologist and the music practitioner. Before the telemusic intervention, the neurologist assessed the patient's diagnoses and their MRIs to see what capacities the patient had and what brain structures had been affected by disease.

### Procedural Methodology

**Figure 1:**
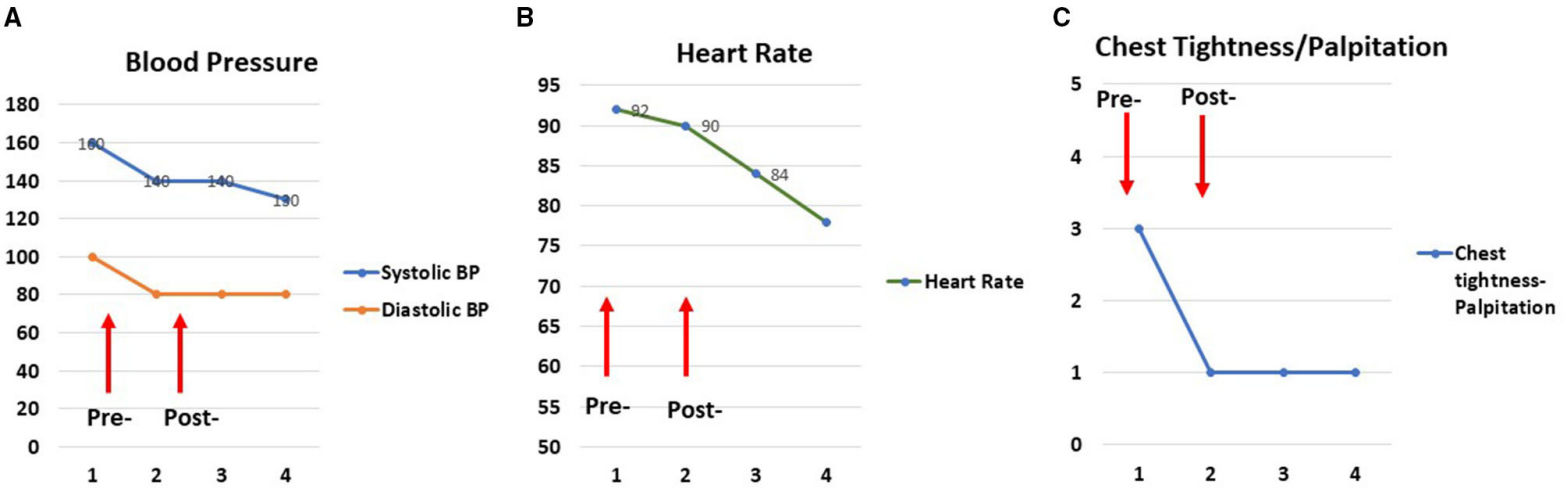
Examples of tension relief following an individual telemusic session. Pre/post and follow-up two measurements of blood pressure **(A)**, heart rate **(B)**, and subjective physical ratings **(C)** for one session show tension relief following a 25-min music intervention in the participant. Two additional measures (numbers 3 and 4) were taken 6 and 12 h after the intervention was completed. The physiologic effects of the music persisted for 12 h.

Before starting the intervention for the hospital patients, the music practitioner intern (MPI), music therapist (MT), and neurologist piloted the use of video-based telemusic interventions to test its effectiveness in a small group of individuals. In a preliminary assessment involving a series of 3 individuals and a total of 17 sessions, we measured blood pressure, heart rate, and respiratory rate before and after clinically designed improvisatory music (CDIM) interventions. CDIM is a type of receptive intervention that possesses 2 strong attributes. (1) It uses strict parameters for rhythm, tempo, range, dynamics, timbre, and silence. (2) This improvisational intervention is live and implemented by a clinically trained musician in which social presence and engagement take place (20). CDIM consists of improvised music with pitches that are within the human vocal range at 131–524 Hz, slow tempi within 50–70 beats per minute, simple rhythms with no syncopation, interspersed with 10–15 s intervals of silence. We measured subjective physical feelings associated with sensations of tension using a 10-point Likert scale. The music intervention was associated with a significant decrease in feelings of stress as measured by decreased blood pressure, heart rate, and physical feelings of COVID-19 related stress, including decreased chest pressure, headache, and facial tension (Figure 1).

The MPI and neurologist met to discuss each patient's condition and what kind of music might be most beneficial to the patient, with input from the patient's MAT. This process was supervised by a licensed music therapist whom they met with periodically. If the patient had a strongly defined MAT assessment and also did not have cognitive impairment, the MPI created a playlist that conformed to the preferences. If the patient was in a delicate and unstable state with pain or confusion, the MPI adjusted the music to aid in regulating vital signs (29) or music that soothed anxiety or pain (30). If the patient was in the epilepsy monitoring unit, the MPI opted for the patient's preferences with improvisation or a complete session of improvisation if the patient seemed exhausted from the testing that takes place in the EMU (31). Most of the music played was slower for all patient conditions, in the 50–70 beats per min tempo. In familiar songs, the melodies were simplified; syncopated, fast, or complicated rhythms were not played (32). On the viola, the MPI restricted improvisation to the lower three strings, playing smooth and soft notes, meandering tonally, while sometimes playing intervals of thirds, fourths, fifths, and sixths. Per patients' remarks, double stop intervals were calming and brought feelings of “expansiveness.”

At the time of the telemusic intervention, the SW placed one of the ward's iPads in the patient's room, so that the patient could easily be viewed by the MPI. The SW called the MPI *via* FaceTime and made additional adjustments for the MPI to best view the patient, in order to be able to monitor the patient for agitation or worsening of condition. Prior discussion of patients with the team was helpful at this point. For patients with hemispatial neglect or hemianopsia, special care was taken to accommodate their condition so the patient could see and pay attention to the screen.

In the telemusic intervention, the MPI introduced herself to the patient. MPI came prepared with a song list or a plan for improvisation. If there was an unusual patient case (e.g., agitated suicidal patient), the team music therapist was consulted to help design the telemusic session. During the telemusic session, the MPI monitored the patient's facial expressions, breathing, and body movements for signs of discomfort or stress. Each session lasted ~30–40 min. On average, 5–8 pieces were played during each session (33). Depending on the patient's condition (e.g., for patients with anxiety or discomfort due to pain) the planned session might be converted to improvisatory music (34).

When the telemusic intervention session ended, the SW entered the patient's room to administer a post-session survey. This survey included 6 statements to which patients responded using a 10-point Likert scale (35) and an open-ended question where patients added any additional feedback or comments. Six scaled statements included the following: (1) The music intervention program improved my mental and emotional state; (2) The music intervention program improved my feelings of tension; (3) The music intervention program improved my feelings of restlessness and/or panic (Including inability to relax and focus, chest discomfort or “butterfly feelings in the stomach,” breathing fast, feeling of impending doom); (4) The music intervention program improved my ability to experience pleasure and contentment; (5) The music intervention program improved my energy level; and (6) I would recommend this music intervention program to others. The survey questions were read to patients and responses were captured in the Sentact platform. Survey results were exported into Excel and analyzed throughout and at the conclusion of the project. Four investigators (CT, BB, DH, MP) evaluated and processed the data and compared results for reliability and reproducibility.

## Results

**Figure 2:**
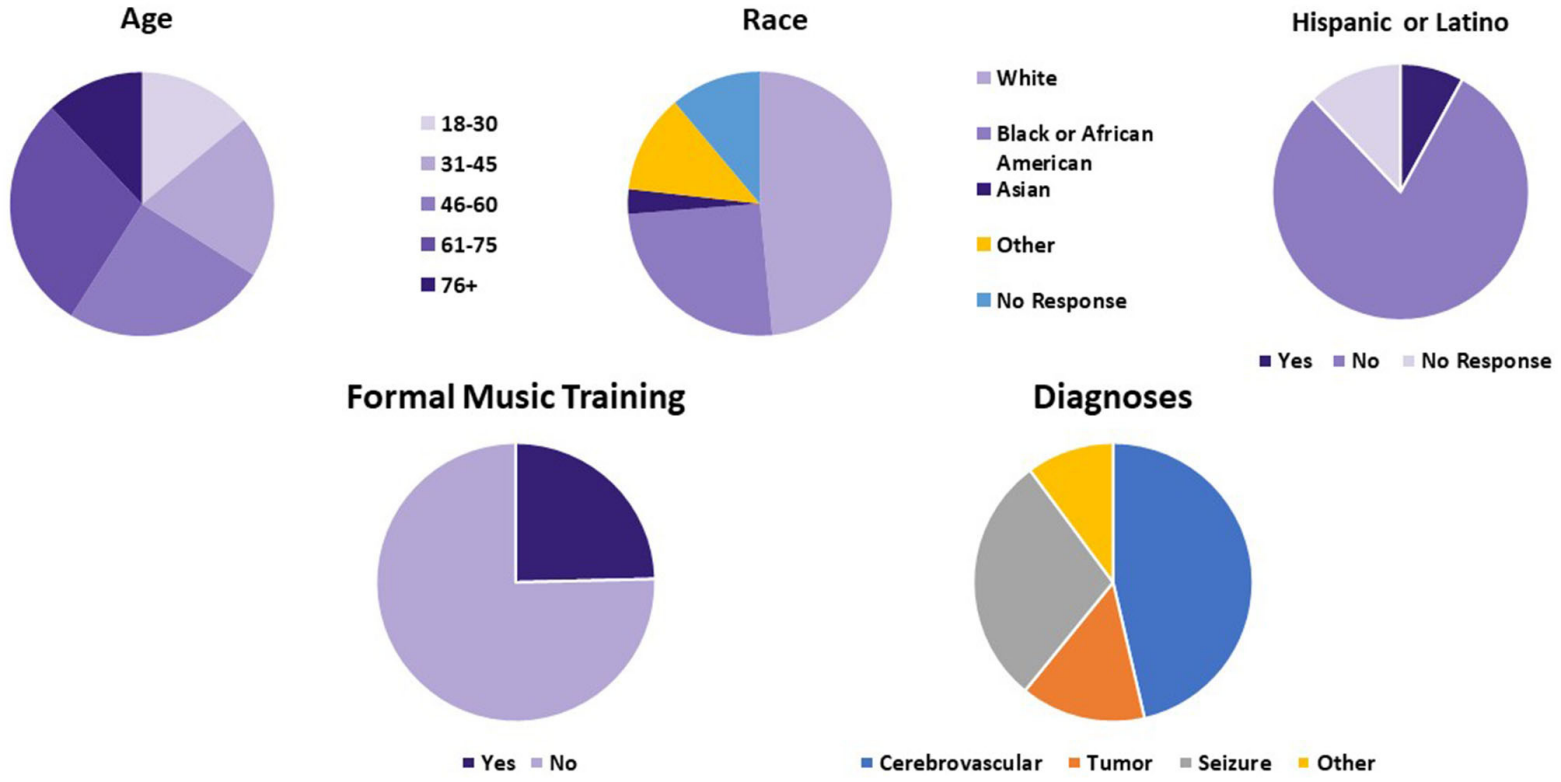
Demographic information based on pre-intervention surveys. Age, diagnosis, ethnic background, and history of formal music training of patients who finished telemusic intervention are demonstrated. NSG, neurosurgical cases.

**Figure 3:**
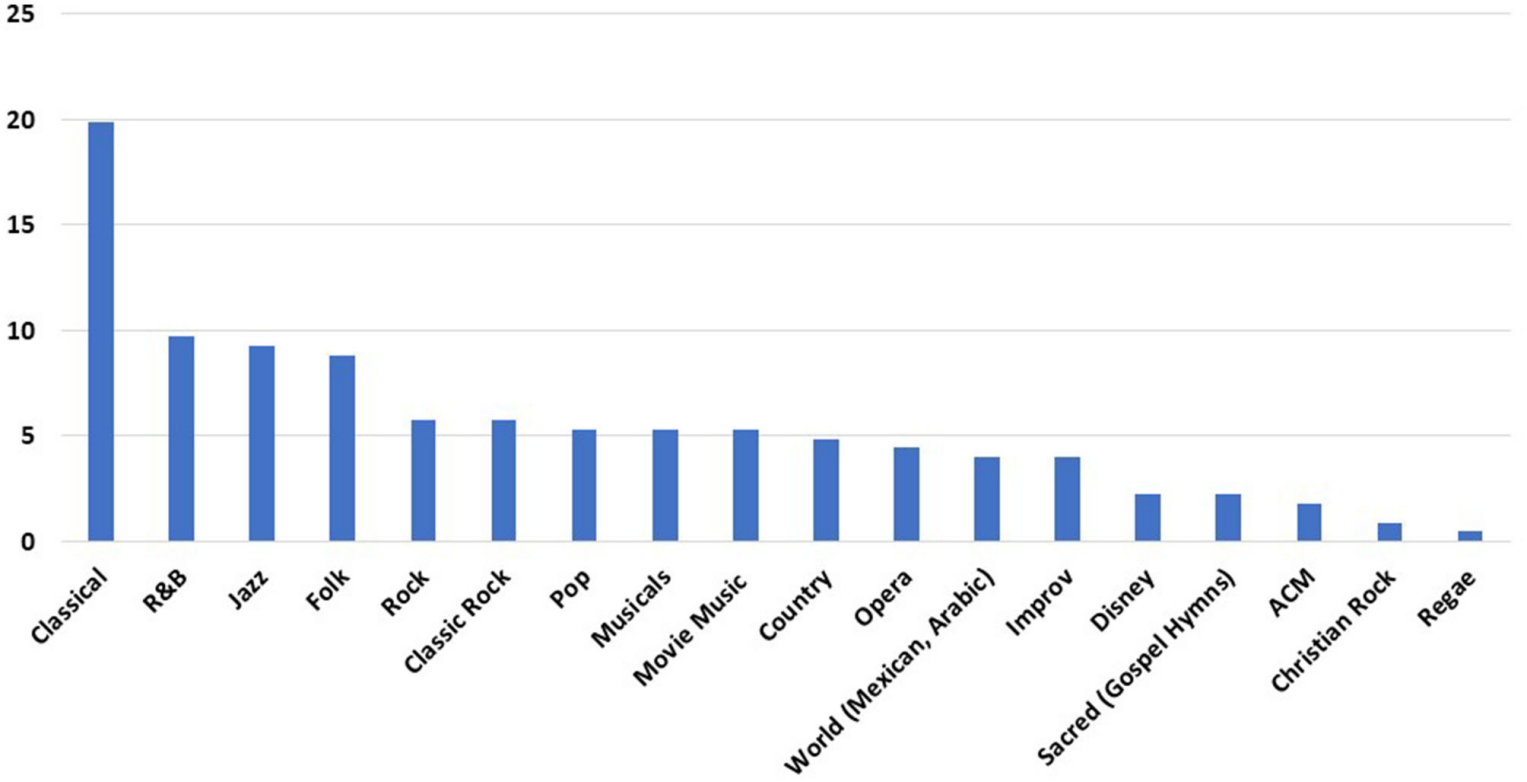
Musical genres used for telemusic intervention. Classical music, Rhythm and Blues (R&B), and Jazz were the top three categories requested by patients. Y-axis depicts how many times each genre was played during 87 sessions of telemusic interventions. ACM, Adult Contemporary Music; Improv, Improvisatory.

During the 3-month period of the intervention, 70% of patients on the neurosciences floor chose to participate. SWs interviewed 126 patients. Telemusic sessions were completed for 87 patients. Reasons for the lack of completion of surveys included early discharge, medical procedures that conflicted with the scheduled telemusic session, and acute change in clinical status requiring medical attention. Few patients changed their minds and chose not to have the music intervention when their scheduled time arrived. Sixty-five patients completed the post-intervention evaluation. Those who did not complete the evaluation had sessions interrupted by medical procedures or had fallen asleep and SWs decided not to disturb them. Distributions for age, gender, ethnicity, diagnosis, and prior music training are represented in Figure 2. Musical genres performed for patients are displayed in Figure 3. Classical, R&B, and Jazz genres constituted the most common genres requested by patients. In 9 cases improvisatory music was utilized based on patients' condition (pain, agitation, discomfort, and fatigue).

**Figure 4:**
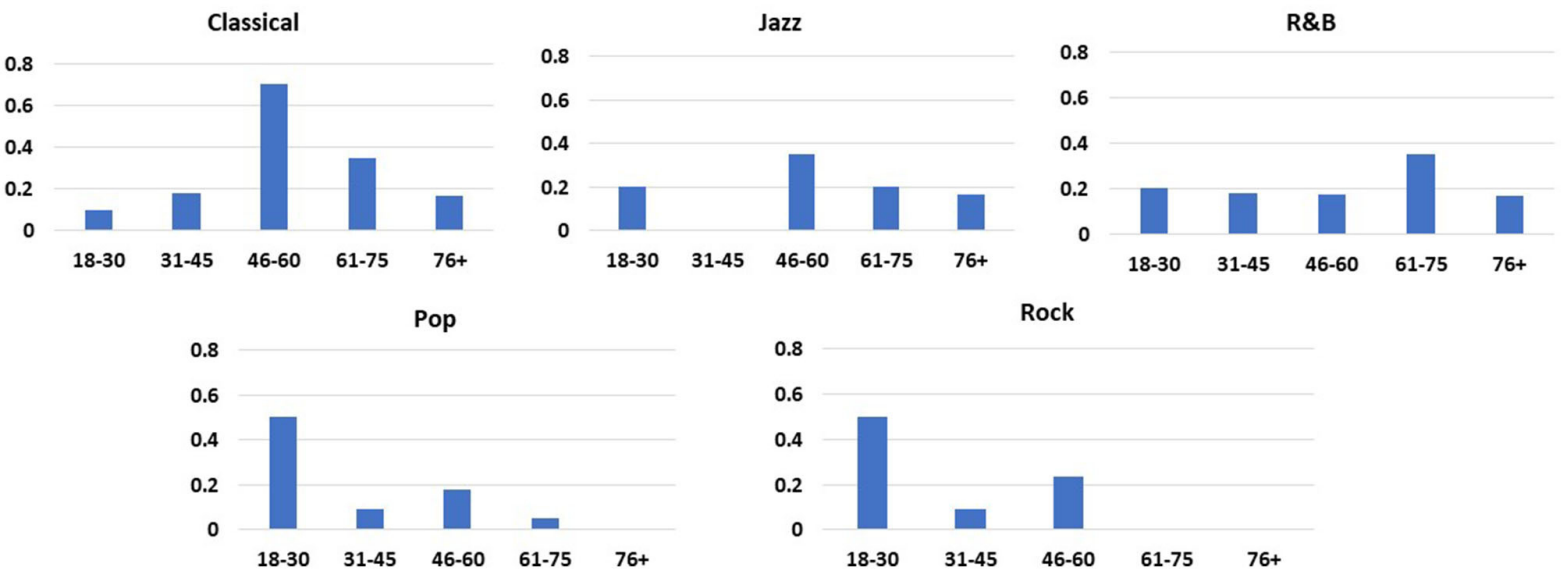
Distribution of music preferences based on age. Classical and Jazz were requested with greater frequency in patients who were 46 above. R&B was the most consistently preferred across all ages. Pop and Rock genres were mostly requested by individuals in the 18–30 years old age range.

Age distribution of genres performed is displayed in Figure 4. Classical and Jazz were requested with greater frequency in patients who were 46 above. R&B was the most consistently preferred across all ages. Pop and Rock genres were mostly requested by individuals in the 18–30 years old age range.

**Figure 5:**
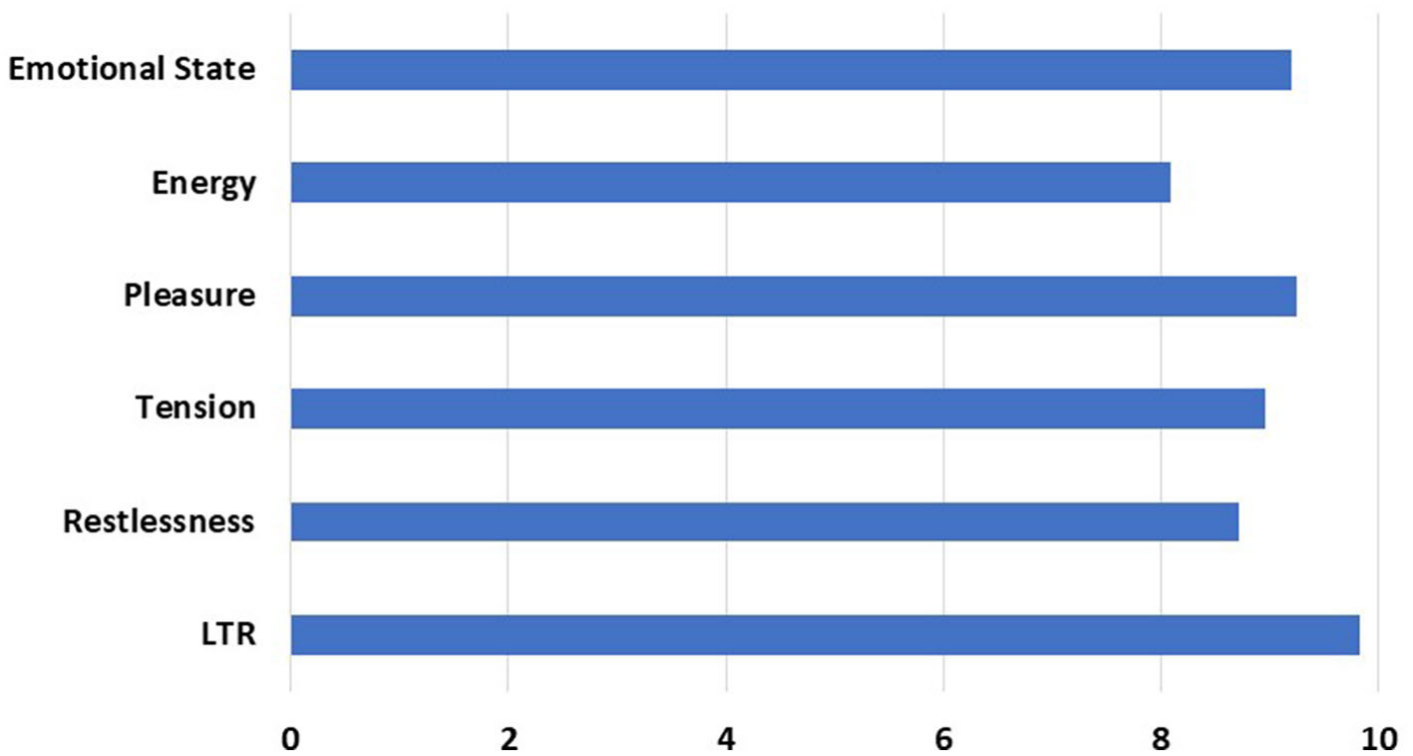
Post-intervention Responses Elicited by Survey. The X-axis represents average Likert scale rating by patients (1–10). Y-axis represents questions asked to measure the effect of the intervention. Patients were assessed for positive change in emotional state, increase in energy level, feelings of pleasure, reduction of tension, and restlessness. LTR, Likelihood to Recommend to others.

In the post-intervention survey, patients were quite satisfied by the intervention citing a likelihood to recommend the program at a rate of 98% based on averaged responses from the Sentact survey (Figure 5). They also reported that the intervention improved their emotional state.

Despite their different degrees of disability, most patients engaged aesthetically with the music being played through facial gestures or by moving both affected and unaffected extremities. Forty-six percent of patients were diagnosed with stroke, 29% had epilepsy, 14% had CNS tumor, and the rest were admitted for surgery or other reasons. Improved emotional state was rated at 92%; experience of pleasure was rated at 92.4%; reduced anxiety was rated at 89.5%.

Some observed responses during the intervention included aesthetic responses from the patient, responding to music by moving their arms or legs including mobile or partially paralyzed extremities. Some patients were in a reduced level of consciousness and yet engaged with the music by arm or leg movements with the tempo or rhythm of the music. Patients with right hemispheric lesions may have difficulty processing music; however, this did not seem to be an obstacle for engaging with music. Patients with left hemispheric lesions and aphasia particularly found music helpful as a non-verbal way of socially engaging. In patients waiting for procedures, music had an especially calming impact, easing anxiety while anticipating their tests or surgeries.

In cases where family members were present during the intervention, family members also reported reductions in stress levels. Family members also found patient engagement with music reassuring. Medical staff additionally reported enhanced patient cooperation following music interventions.

## Discussion

Our 3-month pilot project in the neurosciences unit showed that the telemusic intervention for patients was feasible during the COVID-19 pandemic. Based on our survey, the program had the strongest positive impact on participants' sense of emotional well-being, reduced their tension, and created a sense of pleasure and contentment in an isolated environment governed by the pandemic restrictions. The top music genres used were classical, jazz, and R&B. On average, patients reported a likelihood of recommendation of 98% for the telemusic intervention.

Our results are consistent with in-person hospital-based music interventions. Nayak et al. showed significant improvement in TBI and stroke patients' mood and social interaction following music therapy. Hospital staff also rated participants in music therapy as more actively involved and cooperative in therapy (22). Our experience also confirms Nayak et al.'s observations and our findings were similar with epilepsy, dementia, brain tumor, and neurosurgery patients. Music is known to be used as an adjunct to rehabilitation approaches (14). Our observations demonstrate additional benefits of receptive music interventions to stabilize mood and anxiety in the time of crisis.

Music that was played fit the patients' expectations and preferences, resulting in changes of mood and levels of anxiety. There is little overlap if any between this intervention and the performance of popular songs in their original state. Patients may have listed strong preferences for popular songs that are upbeat with fast tempos, but the musical material was altered in concordance with clinical music therapy principles to bring feelings of calmness and familiarity. Genres most preferred were classical, R and B, and jazz, reflecting the demographic preferences of the patients, since 75% of patients were above 45 years of age. In the youngest age group, the preference was mostly current pop music. For patients with dementia, music they would have been exposed to in their adolescence and early adulthood was selected because this elicited a strong pleasure response (36).

Patients mentioned the difficulty of being in the hospital, not wanting to expose their relatives to COVID-19, and being lonely in addition to struggling with their medical condition. With the pandemic restrictions in the hospital, patients could have one visitor at a time from 9 a.m. to 6 p.m., but many opted not to have visitors to protect their family members from additional risk of infection. Our findings demonstrate the benefit of PFA as shown in other efforts to help individuals affected by the pandemic (1, 37). Ravindran et al. showed that 90% of individuals who connected with a disaster response worker reported satisfaction for such services early on during the COVID-19 pandemic (1). Face-to-face interaction was a critical factor in the initial encounter with the SWs and telemusic intervention. An inpatient study in Wuhan showed that an online intervention without face-to-face interaction was insufficient in delivering PFA to patients hospitalized during the COVID-19 pandemic (38).

Our experience emphasizes the importance of programs enriching hospital time to convert it from a tense experience to an aesthetically meaningful time. This project also provided opportunities for further research into the mechanisms by which music can be of help for admitted neurology patients.

## Limitations

This report was based on qualitative interviews following clinical interventions at the time of crisis. The purpose of this report was to demonstrate feasibility and patient responses during the first months of the COVID-19 pandemic lockdowns. We cannot generalize or comment regarding the degree of efficacy of telemusic intervention as there was no pre/post-evaluation and no control group. However, patients found the telemusic intervention to be a powerful support for them. In addition, our target population was heterogeneous which prevents us from having conclusions about specific neurological conditions. Further research is needed to evaluate the effectiveness of telemusic intervention in a pre/post, controlled design and more homogeneous groups.

## Conclusion

In this paper, we reported the feasibility of a telemusic intervention during the COVID-19 pandemic in an inpatient neuroscience floor at Northwestern Memorial Hospital. During a period of 3 months, 87 patients received the intervention. Based on surveys, a large number of patients found the intervention helpful during their stressful stay while pandemic precautions were in place. Our experience emphasizes the benefits of telemusic interventions as PFA during the unprecedented circumstances of the Coronavirus pandemic. Our further investigations will focus on sustaining such efforts and understanding specific mechanisms by which a sound-based intervention can affect neurologic patients.

## Data Availability Statement

The raw data supporting the conclusions of this article will be made available by the authors upon formal request, without undue reservation.

## Ethics Statement

The studies involving human participants were reviewed and approved by Northwestern University Institutional Review Board. The patients/participants provided their written informed consent to participate in this study.

## Author Contributions

BB: drafting/revision (D&R) of the manuscript, acquisition of data, study concept and design, and analysis and interpretation of data. AM: D&R, study concept or design, and music therapy consultant. SZ, MSh, BS, WH, CBra, and JV: D&R and major role in the acquisition of data. DH: D&R and analysis and interpretation of data. AS, CBre, CR, and MSch: D&R and study design. MP: D&R, major role in the acquisition, and analysis and interpretation of data. KG: D&R, including medical writing for content, and major role in the acquisition of data. DT: D&R, study concept or design, and certified music practitioner. CT: D&R, study concept or design, analysis or interpretation of data, and certified music practitioner intern. All authors contributed to the article and approved the submitted version.

## Funding

This project was funded thanks to Mr. Jeff Hecktman's generous gift to the Department of Neurology. We hereby thank Dr. Dimitri Krainc, Northwestern Feinberg School of Medicine chairman of neurology for his kind support of this project.

## Conflict of Interest

The authors declare that the research was conducted in the absence of any commercial or financial relationships that could be construed as a potential conflict of interest.

## Publisher's Note

All claims expressed in this article are solely those of the authors and do not necessarily represent those of their affiliated organizations, or those of the publisher, the editors and the reviewers. Any product that may be evaluated in this article, or claim that may be made by its manufacturer, is not guaranteed or endorsed by the publisher.
